# From minimally to maximally invasive; VT ablation in the setting of mechanical aortic and mitral valves

**DOI:** 10.1111/jce.15623

**Published:** 2022-07-21

**Authors:** Joshua Hawson, Jonathan Kalman, John Goldblatt, Robert D. Anderson, Troy Watts, Nick Hardcastle, Shankar Siva, Saurabh Kumar, Geoffrey Lee

**Affiliations:** ^1^ Department of Cardiology Royal Melbourne Hospital Parkville Victoria Australia; ^2^ Faculty of Medicine, Dentistry and Health Science University of Melbourne Carlton Victoria Australia; ^3^ Department of Radiation Oncology Peter MacCallum Cancer Centre East Melbourne Victoria Australia; ^4^ Department of Physical Sciences Peter MacCallum Cancer Centre East Melbourne Victoria Australia; ^5^ Department of Cardiology Westmead Hospital and Westmead Applied Research Centre Sydney New South Wales Australia

**Keywords:** ablation, ventricular arrhythmia, ventricular tachycardia

## Abstract

Double mitral and aortic mechanical valves present an access challenge when planning a ventricular tachycardia (VT) ablation. In this case report, we describe a patient who was considered for stereotactic ablative radiotherapy but was unable to proceed due to unfavorable anatomy making them at high risk of fistula formation. The patient went on to have an endocardial VT ablation via mini‐thoracotomy and transapical access without complication. This case highlights the need for careful consideration when planning treatment for patients with double mechanical valves.

## 1 INTRODUCTION

Ablation for ventricular tachycardia (VT) in the setting of mechanical mitral and aortic valves is challenging, as entry into the ventricle via traditional methods is not possible. In addition, epicardial ablation is often challenging due to pericardial adhesions resulting from the initial operation. Alternate approaches for ablation have therefore been developed, including direct ventricular puncture.^1^ More recently stereotactic ablative body radiotherapy (SABR) has been described as an alternate, noninvasive method for VT ablation.^2^

## 2 CASE

A 52‐year‐old male presented with recurrent VT requiring multiple episodes of antitachycardia pacing (ATP) and shocks. His past history included a bileaflet mechanical aortic valve and caged ball mechanical mitral valve, inserted in 1983 for rheumatic heart disease, and nonischemic cardiomyopathy. There was long‐standing left hemidiaphragm paralysis following the initial valve replacement operation. An implantable cardiac defibrillator was initially inserted in 2007 for primary prevention, with subsequent appropriate shocks for VT in 2014. More recently he had suffered increasing episodes of VT despite escalating medical therapy. Antiarrhythmic therapy at the time of presentation included amiodarone, mexiletine, and bisoprolol.

Echocardiography revealed a left ventricular (LV) ejection fraction of 30%–35%, with the distal two‐thirds of the ventricle hypokinetic and thinned. Computerized tomography (CT) coronary angiogram demonstrated no significant coronary artery disease. Device interrogation revealed 21 episodes of VT in the preceding month with 8 episodes of ATP. Twelve‐lead ECG of the clinical VT demonstrated a right bundle branch block pattern in V1, late transition (V5), deeply negative polarity in leads I and aVL, and positive polarity lead III. The clinical VT localized to segment 6 of the 17‐segment American Heart Association heart model according to contemporary ECG criteria.^3^

**Figure 1:**
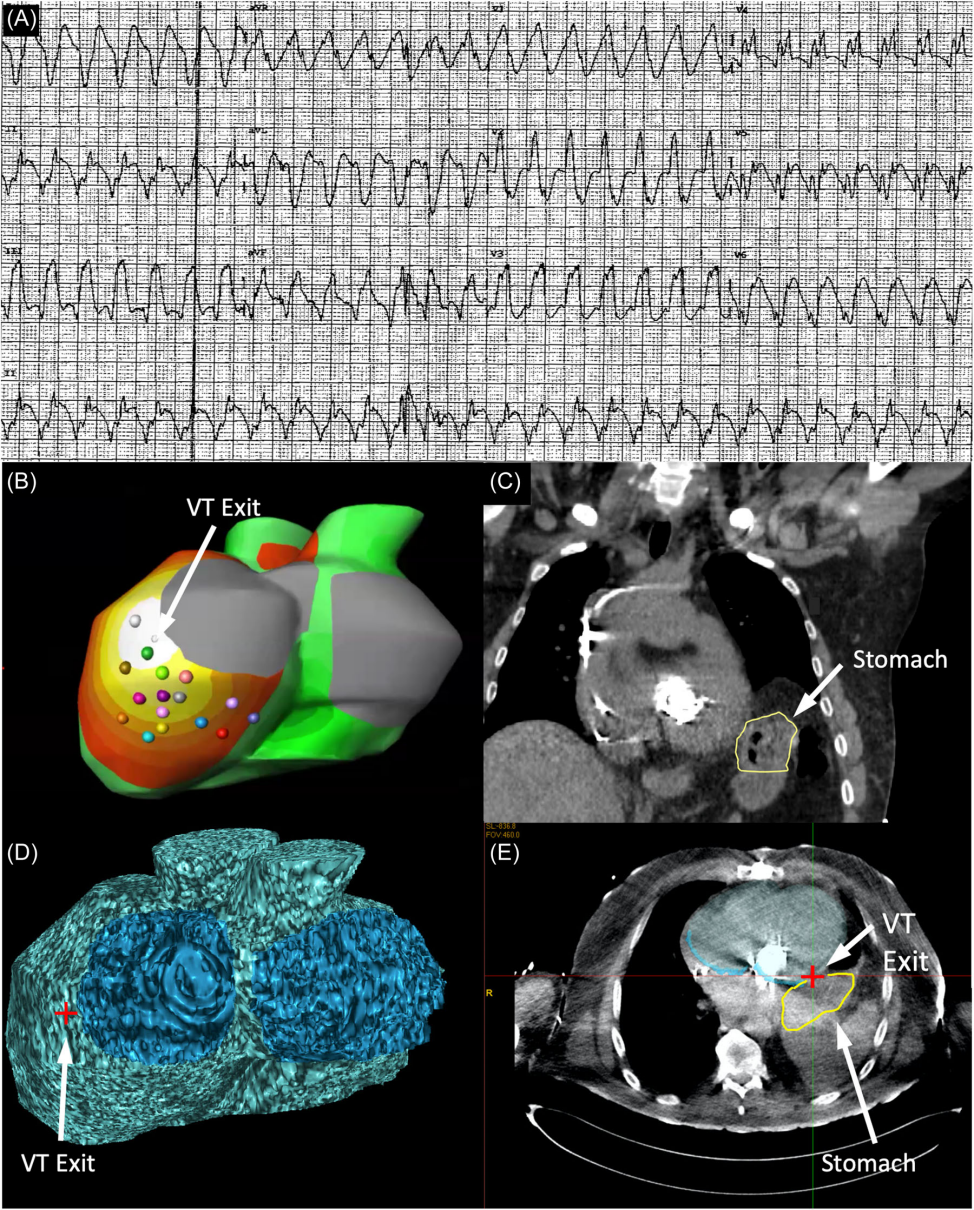
Planning for stereotactic ablative body radiotherapy. (A) Twelve‐lead ECG of clinical VT. (B) ECGI activation map demonstrating earliest activation in the basolateral region of the LV. (C) Coronal slice CT demonstrating stomach (outlined in yellow) abutting the basolateral LV wall due to an elevated left hemidiaphragm. (D) Fused CT/ECGI demonstrating VT exit site (red cross) with the corresponding location on CT (Panel D, red cross), directly adjacent to the stomach. CT, computerized tomography; ECGI, electrocardiographic imaging; LV, left ventricular; VT, ventricular tachycardia

Given the dual mechanical valves and LV dysfunction, the patient was considered a candidate for a SABR VT ablation. Mapping with noninvasive electrocardiographic imaging demonstrated a VT exit from the basolateral LV. Unfortunately, due to the raised left hemidiaphragm, this area directly abutted the stomach on the fused CT (Figure 1). After consideration, it was deemed that the risk of a gastroventricular fistula was too high to proceed with SABR.

**Figure 2:**
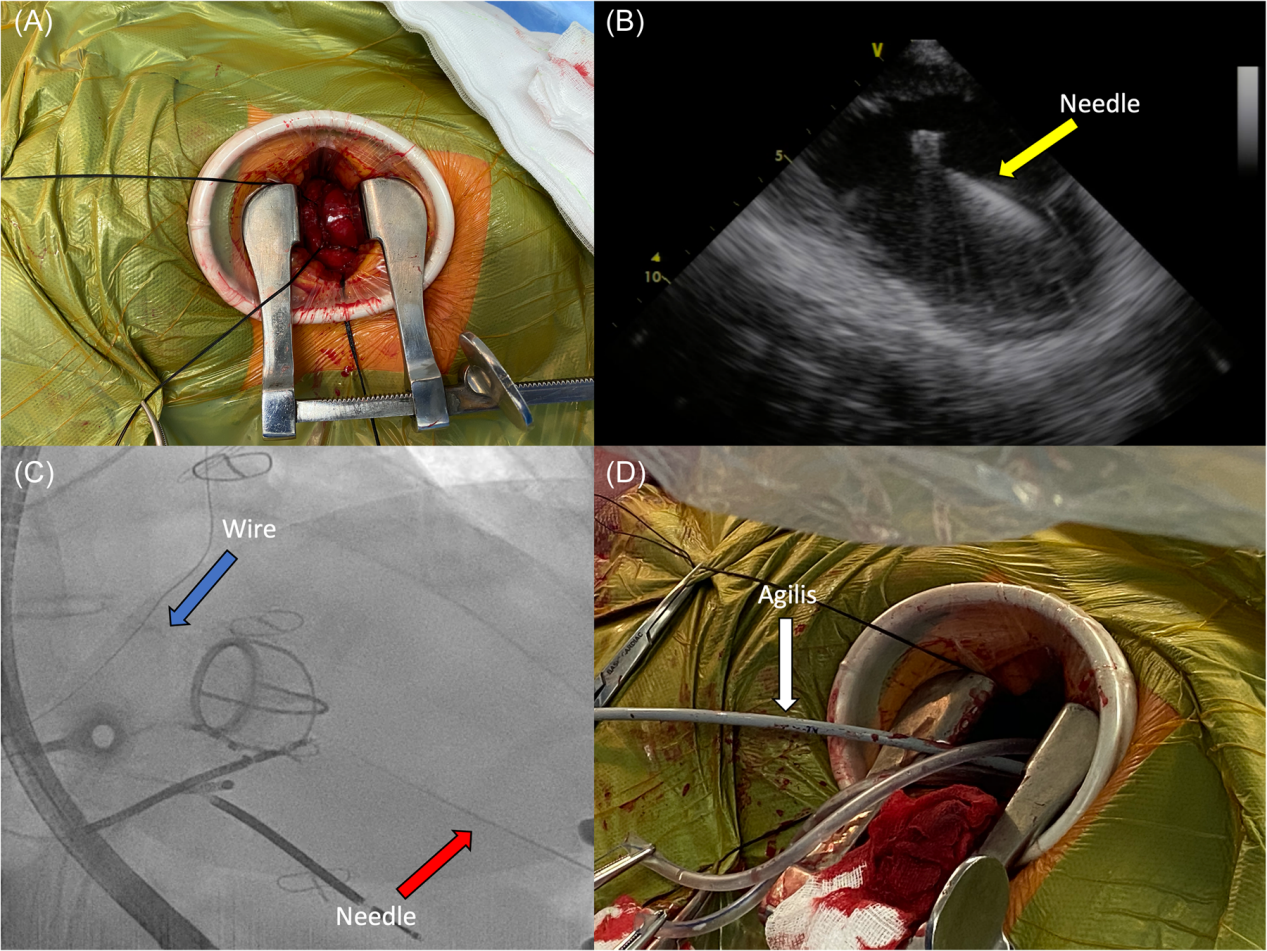
Surgical transapical access. (A) Mini‐thoracotomy revealing cardiac apex. (B) ICE image of apical puncture with a needle (white arrow). (C) Fluoroscopic image of apical puncture with a needle (red arrow) and guidewire advanced through the ball‐and‐cage mechanical valve into the left atrium (blue arrow). (D) Agilis EPI sheath in position through apical puncture (white arrow). ICE, intracardiac echocardiography

**Figure 3:**
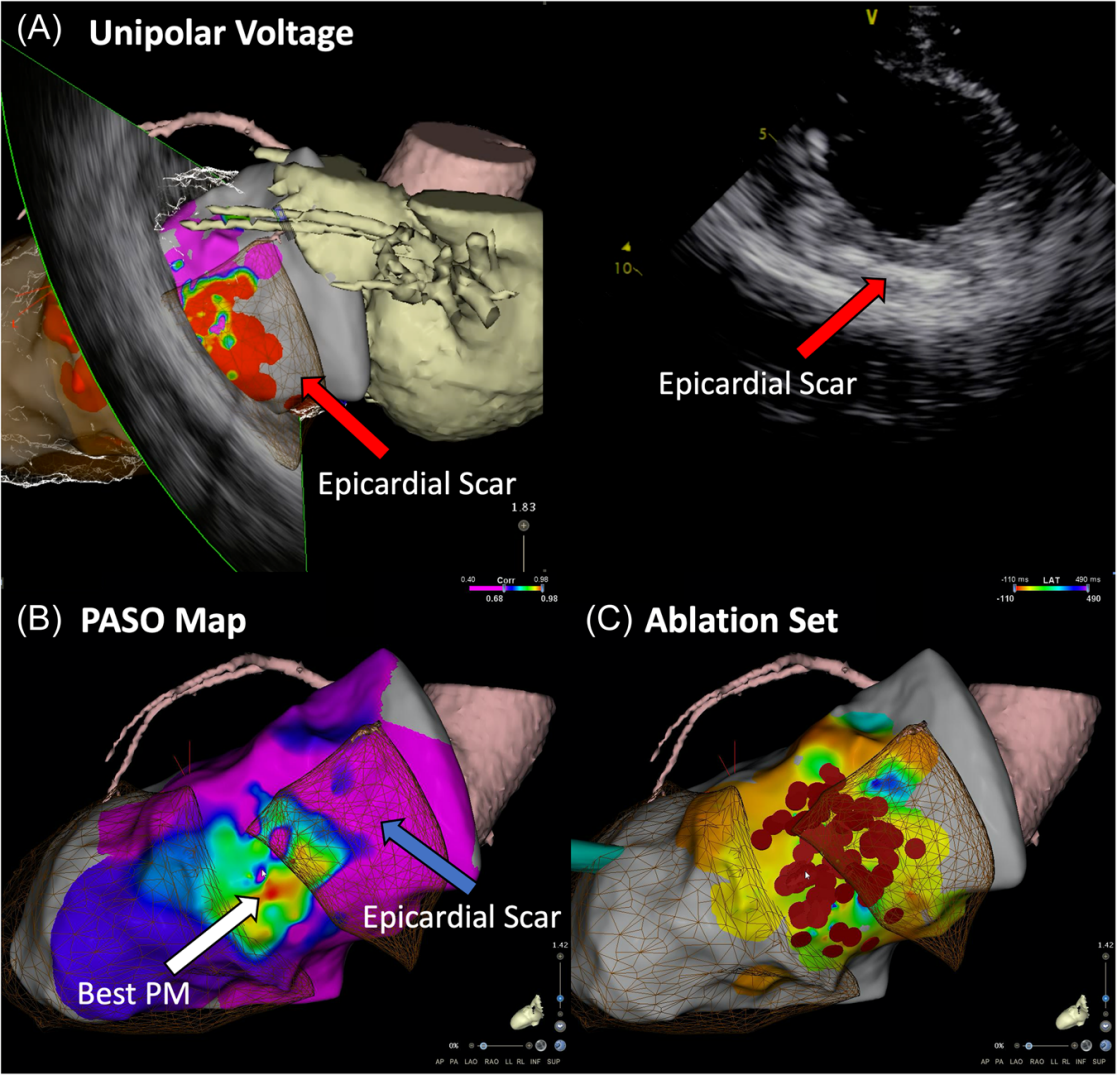
Ablation procedure. (A) ICE image of lateral scar (red arrow) with corresponding 3D CARTO map. (B) Map of pacing sites, demonstrating best match at border‐zone of basal scar (mesh area labeled with blue arrow). (C) Final ablation set over the mid‐basal lateral wall. ICE, intracardiac echocardiography

An endocardial VT ablation via a transapical approach was subsequently performed. Femoral venous access was obtained for hexapolar His and decapolar coronary sinus catheters. Intracardiac echocardiography (ICE) was used to create a three‐dimensional (3D) shell before apical puncture. Segmentation of the myocardial scar was performed using the CARTOSOUND module. This demonstrated extensive transmural scar in the apical region, with an epicardial scar in the basolateral LV. The apex was accessed via mini‐thoracotomy. Access to the pericardial space was attempted but impossible due to extensive adhesions. Under fluoroscopic and ICE guidance, the apical puncture was performed using a standard Cook access needle, with the advancement of a guidewire. An Agilis EPI steerable sheath was then advanced into the LV cavity using the Saldinger technique (Figure 2). A voltage map of the lateral LV was created with the ablation catheter, demonstrating predominantly low unipolar voltage, consistent with the epicardial scar seen on ICE (Figure 3).

The clinical VT was not inducible despite programmed stimulation, both with and without isoprenaline. Multiple nonclinical VTs were induced that were hemodynamically untolerated and required cardioversion. A substrate‐based approach using signal analysis and pacemapping was therefore performed. Due to the mini‐thoracotomy, leads V3–V6 were two intercostal spaces lower than the standard position. With this consideration, the best pacemap was obtained in the mid‐lateral LV. Ablation at this site caused the onset and termination of VT. Thereafter, homogenization of the scar was performed, targeting late potentials (LP) and local abnormal ventricular activations (LAVA). Over 75 min of ablation was performed using 0.45% saline irrigation at 40 W. Subsequent mapping of the LV apex revealed no LPs or LAVA as ablation targets. VT was noninducible at the end of the procedure on repeat programmed stimulation. There were no procedural complications. At 1‐month follow‐up, the patient had 11 episodes of nonsustained VT but had not required readmission to the hospital.

## 3 DISCUSSION

This report describes a complex management case in a patient with recurrent VT despite escalating medical therapy. Patients with mechanical aortic and mitral valves present a challenging management case in the setting of recurrent VT, as traditional retrograde and anterograde access approaches are not possible. Historically labeled a “no‐entry” ventricle, various methods have been described for ablation in this setting.

Pioneered by Cuculich et al., SABR provides a promising alternative means for delivering ablation in a no‐entry ventricle.^2^ ECGI was performed on our patient with a plan to proceed to SABR. In the limited published data, SABR has a favorable short‐term safety profile. However, due to the raised left hemidiaphragm, the stomach directly abutted the target area. A systematic review by van der Ree et al. demonstrated that no major complications occurred within the perioperative or early follow‐up following SABR VT ablation.^4^ However, long‐term follow‐up of the ENCORE‐VT study reported two late complications, the most serious of which being a gastropericardial fistula.^5^ Given the close proximity of the target area and the stomach, the risk of late fistula formation was considered prohibitively high.

Other strategies for ablation in the setting of a no‐entry ventricle include (1) direct puncture of the interventricular septum, (2) puncture through the ventricular free wall, (3) transapical puncture, (4) transcoronary ethanol ablation.^1^, ^6^, ^7^ Recently, Santangeli et al. described puncture from the right atrium into the inferior septal process of the LV to gain access to the LV cavity for ablation.^8^ Given the left basolateral origin of the VT in our patient, and the presence of a caged ball mitral valve, this method was deemed inappropriate due to the angle of approach. An apical approach was considered the most appropriate option; given the association of percutaneous LV puncture with several complications, including hemorrhage and coronary artery puncture, this was performed via a mini‐thoracotomy with surgical support.^1^

Consideration needs to be made when performing an apical puncture, either transcutaneously or via a mini‐thoracotomy. In the setting of tilting disc valves, advancement of guidewires and sheaths creates a risk of valve entrapment and acute regurgitation, which can be fatal.^9^ In this case, the presence of a caged ball valve in the mitral position granted a suitable target for the guidewire, as acute entrapment of a caged ball valve is of negligible risk. In previous descriptions, the Cook needle was connected to pressure monitoring to determine when access to the LV cavity had been gained.^10^ In this case, the puncture was performed under fluoroscopic and ICE guidance, allowing the needle to be pre‐loaded with the guidewire, minimizing blood loss and streamlining access.

In the absence of inducible VT for mapping, a substrate‐based approach is mandated. The use of ICE with CARTOSOUND (Biosense Webster) proved a useful tool in this case. In addition to creating a 3D shell geometry before puncture, ICE also identified a scar in the area of interest, which was not previously seen on the preprocedural echocardiogram or CT. The ability to define a subendocardial macroreentrant VT circuit with pacemapping has previously been described by de Chillou et al.^11^ In this case, pacemapping revealed a centrifugal pattern of accuracy, likely representing an endocardial breakout of an intramural/epicardial circuit. Given the epicardial circuit, as suggested by the pacemapping and ICE‐defined epicardial scar, ablation with 0.45% saline irrigation was performed. This has been demonstrated in animal models to produce deeper ablation lesions and has been evaluated prospectively for targeting deep myocardial substrates.^12^, ^13^ Ablation lesions of up to 120 s were delivered to maximize lesion depth.^14^

Following ablation, a box suture was used for apex hemostasis on sheath removal with good effect. Transcutaneous approaches have previously described using percutaneous vascular occlusion devices as an alternate means of hemostasis. One attempt to puncture the apex without a method of active hemostasis following sheath removal has been described, resulting in near‐fatal hemorrhage.^10^ This approach was used historically during diagnostic procedures, when measuring the LV pressure in the setting of a stenosed aortic valve, but should not be adopted when using larger sheath sizes in therapeutic procedures.^15^

## 4 CONCLUSION

Whilst multiple approaches have been developed to treat VT in the setting of a no‐entry ventricle, the data for each of these remains limited. In this case, direct apical puncture via mini‐thoracotomy provided a safe and effective approach. This case highlights the fact that the noninvasive approach is not always the safest, and caution needs to be exercised when planning a treatment strategy.
